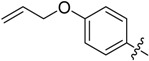# Design, Synthesis, and Biological Activity of Tetrahydrobenzo[4,5]thieno[2,3-*d*]pyrimidine Derivatives as Anti-Inflammatory Agents

**DOI:** 10.3390/molecules22111960

**Published:** 2017-11-13

**Authors:** Yuan Zhang, Lu Luo, Chao Han, Handeng Lv, Di Chen, Guoliang Shen, Kaiqi Wu, Suwei Pan, Faqing Ye

**Affiliations:** 1 School of Pharmaceutical Sciences, Wenzhou Medical University, Wenzhou 325035, China; zy8428@126.com (Y.Z.); 15868535718@163.com (L.L.); hannoverkalte@163.com (C.H.); lhd19924@163.com (H.L.); 15958709517@163.com (D.C.); shen09427@163.com (G.S.); 18268253895@163.com (K.W.); psw835224635@126.com (S.P.); 2 Department of Forensic Medicine, School of Medicine, Xi’an Jiaotong University, Xi’an 710061, China

**Keywords:** tetrahydrobenzo[4,5]thieno[2,3-*d*]pyrimidine, anti-inflammatory, cytotoxicity, paw edema

## Abstract

We designed and synthesized 26 prototype compounds and studied their anti-inflammatory activity and underlying molecular mechanisms. The inhibitory effects of the compounds on the production of nitric oxide (NO), cytokines, inflammatory-related proteins, and mRNAs in lipopolysaccharide (LPS)-stimulated macrophages were determined by the Griess assay, Enzyme linked immunosorbent assay (ELISA), Western blot analysis, and Reverse transcription-Polymerase Chain Reaction (RT-PCR), respectively. Our results indicated that treatment with **A2**, **A6** and **B7** significantly inhibited the secretion of NO and inflammatory cytokines in RAW264.7 cells without demonstrable cytotoxicity. It was also found that **A2**, **A6** and **B7** strongly suppressed the expression of inducible nitric oxide synthase (iNOS) and cyclooxygenase enzyme COX-2, and prevented nuclear translocation of nuclear factor κB (NF-κB) p65 by inhibiting the degradation of p50 and IκBα. Furthermore, the phosphorylation of mitogen-activated protein kinase (MAPKs) in LPS-stimulated RAW264.7 cells was significantly inhibited by **A2**, **A6** and **B7**. These findings suggest that **A2**, **A6** and **B7** may operate as an effective anti-inflammatory agent through inhibiting the activation of NF-κB and MAPK signaling pathways in macrophages. Moreover, rat paw swelling experiments showed that these compounds possess anti-inflammatory activity in vivo, with compound **A6** exhibiting similar activities to the reference drug Indomethacin.

## 1. Introduction

Activated macrophages play crucial roles in the initiation and maintenance of inflammation. Following activation by inflammatory stimuli such as lipopolysaccharide (LPS), macrophages secrete a number of potent bioactive inflammatory mediators, including nitric oxide (NO), prostaglandins (PGs), leukotrienes (LTs), and cytokines such as interleukin-1β (IL-1β), IL-6, and tumor necrosis factor α (TNF-α), that contribute to the activation of mitogen-activated protein kinase (MAPK) and nuclear factor κB (NF-κB) [1,2,3,4,5,6,7,8,9,10]. Of note, as an important inflammatory mediator, the paradoxical role of NO in the pathogenesis of inflammation generally depends upon concentration [11]. Appropriate levels of NO produced by inducible NO synthase (iNOS) in response to inflammatory stimuli such as interferon-γ (IFN-γ), IL-1β, and LPS assist in mounting an effective defense against pathogens [12]. However, sustained overproduction of NO by iNOS is believed to be detrimental to the host and is associated with the pathogenesis of a variety of inflammatory disorders [13]. Therefore, pharmacological interference with NO production is appreciated as a promising strategy of therapeutic intervention in inflammatory diseases [14]. Conversely, while the major role of housekeeping cyclooxygenase enzyme COX-1 is to regulate the arachidonic acid metabolic pathway [15], the inducible cyclooxygenase COX-2 is responsible for the production of pro-inflammatory prostaglandins [16,17], which makes COX-2 a selected target of anti-inflammatory drugs. Starting from the clinical use of aspirin, diclofenac, and indomethacin as non-steroidal anti-inflammatory drugs (NSAIDs) [18,19], the development of COXIBs [20,21,22] has provided some relief to patients suffering from a wide spectrum of inflammatory diseases. However, the cardiovascular side effects associated with the use of COXIBs is a limiting factor that has subdued the medicinal applications of this class of anti-inflammatory drugs [23,24,25,26,27,28], and hence, the search for new anti-inflammatory chemical entities continues.

**Scheme 1:**
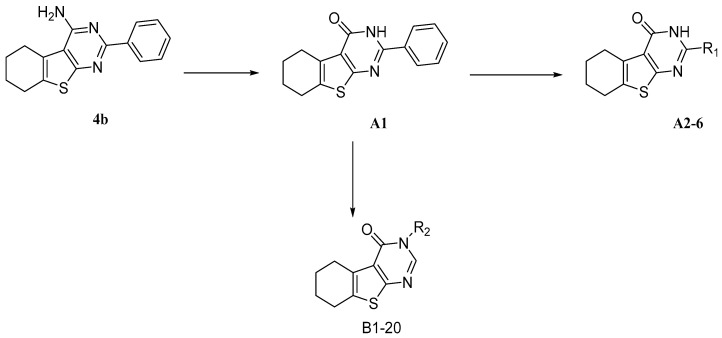
Design of tetrahydrobenzo[4,5]thieno[2,3-*d*]pyrimidine derivatives.

Previously, during the study of tetrahydrobenzo[4,5]thieno[2,3-*d*]pyrimidine series of fibroblast growth factor FGFR1 inhibitors, we were intrigued by the findings that compounds such as **4b** (Scheme 1) inhibited the production of TNF-α and IL-6 [29]. In the literature, it is known that compounds with a similar thieno[2,3-*d*]pyrimidine scaffold has been reported as anti-inflammatory agents [30,31]. In addition, as a bioisostere of quinazoline, thieno[2,3-*d*]pyrimidine has been used extensively for a pharmacophore and synthesis of compounds with diverse biological activities, including anti-tumor [32,33,34,35,36,37,38], anti-microbial [33,39], anti-viral [40,41,42], anti-diabetic [43], anti-anxiolytic [44], and antioxidant [45] activities. We therefore set out to explore the potential anti-inflammatory activity of **4b**, the tetrahydrobenzo[4,5]thieno[2,3-*d*]pyrimidine compound; however, the activity of **4b** has room for improvement, so we attempted to change the amino into carbonyl to obtain the compound **A1**, possessing hydrogen bond receptor to improve the binding between compounds and inflammatory cytokine. According to preliminary anti-inflammation activity screening, A1 is superior to **4b** (51.64% and 49.83% for anti-IL-6, as well as 50.70% 48.23% for anti-TNF-alpha). Furthermore, we performed further structural modifications to **A1** at 2 and 3 positions, respectively, to get compounds with better anti-inflammatory activity and to study the underlying molecular mechanisms of this novel class of compounds.

## 2. Results and Discussion

### 2.1. Chemistry

**Scheme 2:**
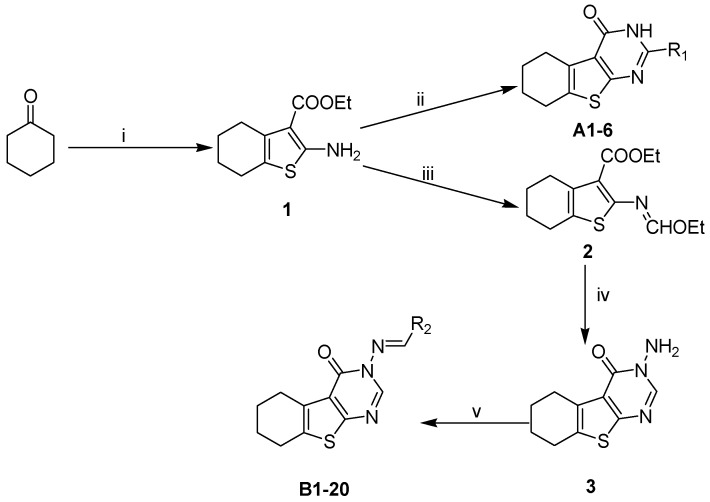
Synthesis of target compounds. Conditions: (**i**) S, Et_2_NH, EtOH, NCCH_2_COOEt; (**ii**) R_1_CN, HCl, dioxane, EtOH; (**iii**) Ac_2_O, CH(OEt)_3_; (**iv**) NH_2_NH_2_·H_2_O; and (**v**) R_2_CHO, EtOH, AcOH.

Based on the structure of template **4b**, we envisaged that it would be interesting to explore the optimal interaction between the phenyl substituent and its biological target, as well as to exploit any potential hydrogen bonding that could result from the amino group on the pyrimidine ring. Thus, we attempted to introduce a carbonyl in place of the amino group, and to modify the 2-, and 3-positions of the tetrahydrobenzo[4,5]thieno[2,3-*d*]pyrimidine system. The construction of the desired template that features a fused tricyclic heterocycle began from the condensation of cyclohexanone and ethyl cyanoacetate in the presence of elemental sulfur (Scheme 2). The key intermediate **1** has functionalities at the 2- and 3-positions of the tetrahydrobenzothiophene ring that allow formation of the pyrimidine ring with different substituents. In one route, reaction of intermediate **1** with aryl or alkyl nitrile generated compounds **A1**–**6** in good yields (Table 1). Alternatively, reaction of **1** with triethyl orthoformate in reflux acetic anhydride provided intermediate **2**, which upon treatment with hydrazine hydrate readily cyclized to give compound **3**. The exocyclic amine in **3** was then further functionalized to provide a variety of hydrazine imines, **B1**–**20** (Table 2), using selected aldehydes.

### 2.2. Biological Studies

#### 2.2.1. Cytotoxicity Assay

**Figure 1:**
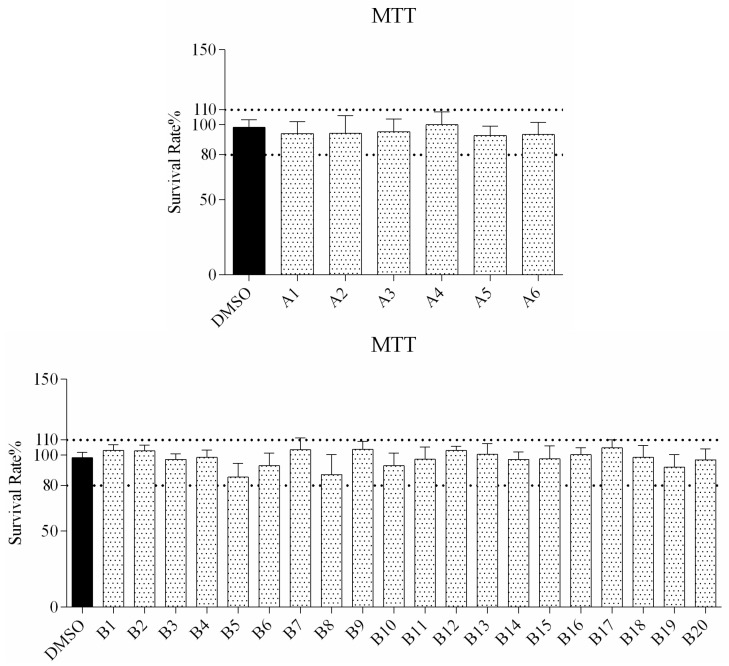
In vitro survival rate of RAW264.7 cells treated with compound **A1**–**6** and **B1**–**20** at 160 μM.

The cytotoxicity of all compounds was evaluated in RAW264.7 cells by MTT assay after 72 h of treatment. As observed from the cell viability data in Figure 1, the survival rate of all compound treated cells is higher than 80%, indicating that a concentration of up to 160 μM was not associated with any significant change in overall cell viability.

#### 2.2.2. Effects of Compound Treatment on Inflammatory Cytokine Secretion

**Figure 2:**
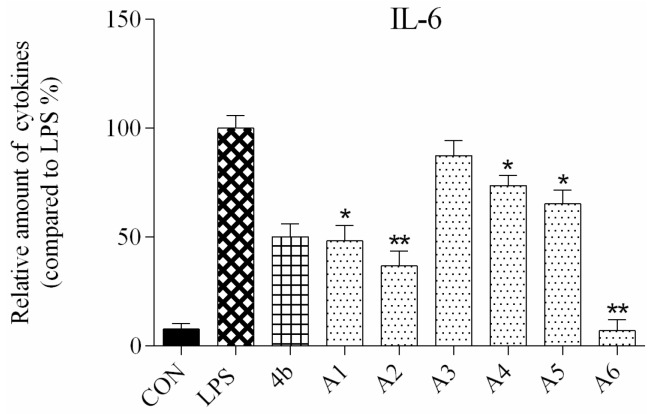

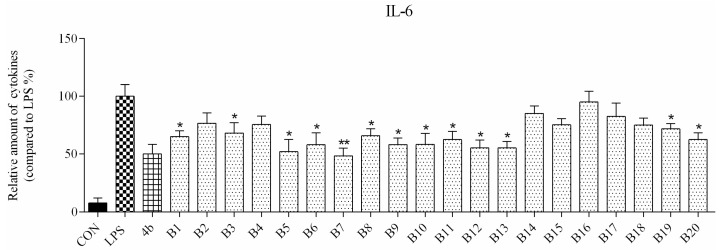
Relative levels of cytokine IL-6 in macrophages after treatment with compounds **A1**–**6** and **B1**–**20** at 10 μM concentration as compared to LPS group and indomethacin group (*: *p* < 0.05; **: *p* < 0.01).

**Figure 3:**
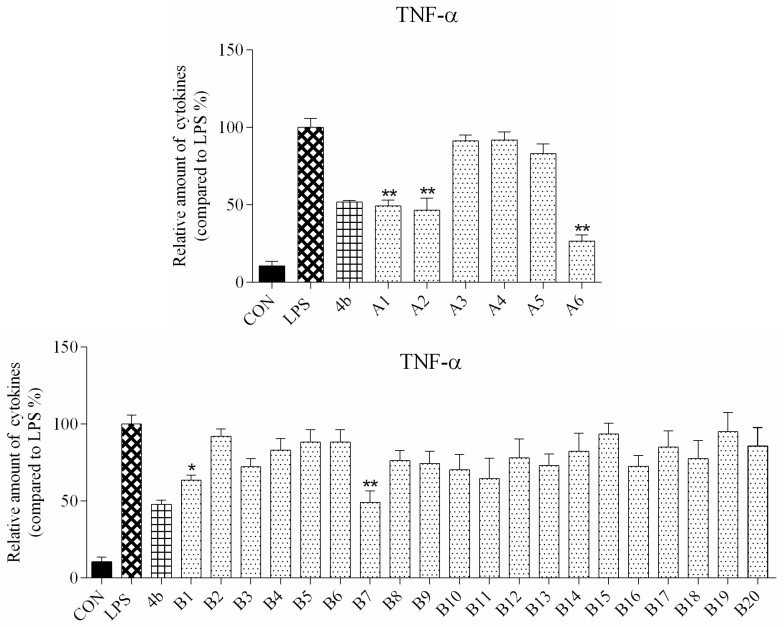
Relative levels of cytokine TNF-α in macrophages after treatment with compounds **A1**–**6** and **B1**–**20** at 10 μM concentration as compared to LPS group and indomethacin group (*: *p* < 0.05; **: *p* < 0.01).

ELISA was used to evaluate secretion of the cytokines TNF-α and IL-6 in macrophages following compound treatment. As shown in Figure 2 and Figure 3, the secretion of inflammatory cytokines was significantly inhibited by most of the compounds at 10 μM concentration. Notably, compounds **A2**, **A6**, and **B7** most strongly suppressed secretion of TNF-α and IL-6, and were selected for further characterization.

#### 2.2.3. Preliminary Structure Activity Relationship (SAR)

**Figure 4:**
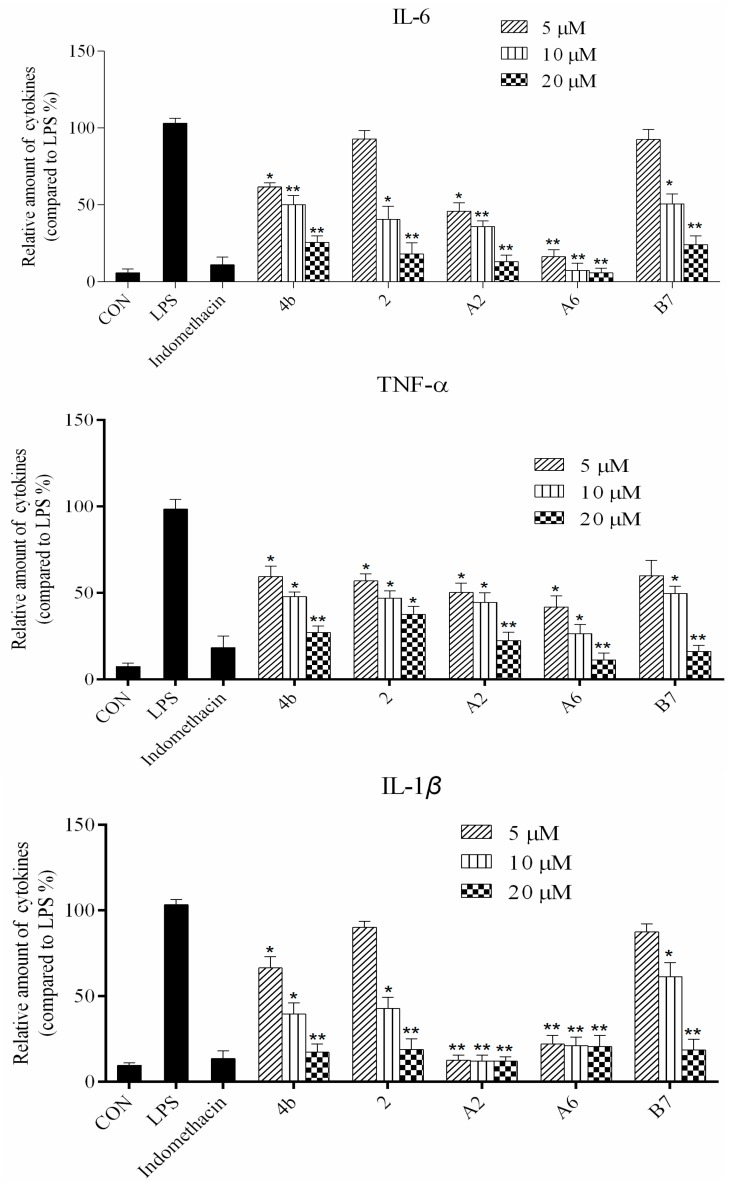

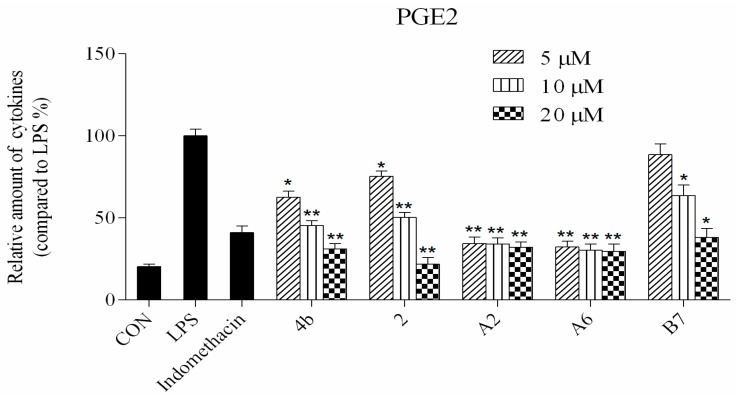
Compounds **4b**, **2, A2, A6**, and **B7** reduced the production of IL-6, TNF-α, IL-1β, and PGE2 at concentrations of 5 μM, 10 μM, and 20 μM (*: *p* < 0.05; **: *p* < 0.01).

As shown in Figure 2, all compounds had significantly low cytotoxicity. As shown in Figure 3 and Figure 4, most of the tetrahydrobenzo[4,5]thieno[2,3-*d*]pyrimidine derivatives were found to exhibit comparable anti-IL-6 and anti-TNF-alpha activity with **4b**, especially **A2**, **A6**, and **B7**, which showed great anti-inflammation activity. Replacing with chloromethyl group and pyridine at 2 position obtained better activity than **4b** and other substitutes; the two groups, as electron donating groups, can improve the anti-inflammation activity. Moreover, replacing with naphthyl group at 3 position obtained the better activity for compound **B7**; naphthyl group can offer multi-π–π conjugated system, which contributes to binding with inflammatory cytokine obtain better inhibitory effection.

#### 2.2.4. Dose Response Effects of Compound **A2**, **A6**, **B7** on Inflammatory Cytokine Secretion

ELISA was used to evaluate cytokine TNF-α, IL-6, IL-1β, and PGE2 secretion in macrophages following treatment with compounds **4b**, **2**, **A2**, **A6**, and **B7**. As shown in Figure 4, the secretion of four inflammatory cytokines was significantly inhibited by compounds **4b**, **2**, **A2**, **A6**, and **B7** in a dose-dependent manner. Notably, treatment with **A2** and **A6** strongly suppressed cytokine secretion more significantly than compounds **4b**, **2**, and **B7**. Moreover, compound **A6** inhibited the production of four inflammatory cytokines with similar activity to the positive control indomethacin at 10 μM.

#### 2.2.5. Inhibitory Effects of Compounds **A2**, **A6**, **B7** on NO Production

**Figure 5:**
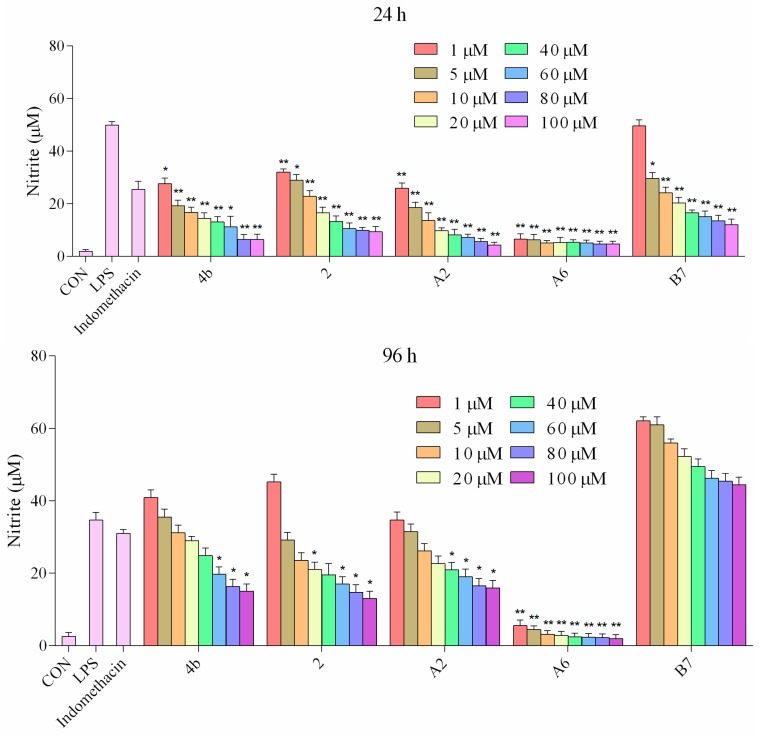
Production of NO in macrophages following treatment with different concentrations of **4b**, **2**, **A2**, **A6**, **B7**, and 1 μM indomethacin for 24 and 96 h (*: *p* < 0.05; **: *p* < 0.01).

It has been well established that NO production is correlated with various inflammatory diseases. We investigated the suppressive effects of compounds **4b**, **2**, **A2**, **A6**, and **B7** on NO levels in macrophages stimulated with LPS. The supernatant was treated with a range of concentrations (1–100 μM) of compounds for 1 h followed by stimulation with LPS for 24 h and 96 h, and NO production was measured using Griess reagent. It was found that compounds **4b**, **2**, **A2**, **A6**, and **B7** dramatically inhibited the release of NO in a dose-dependent manner following LPS stimulation (Figure 5). The effects at 24 h were more significant compared to 96 h. In particular, compound **A6** displayed higher inhibitory activity when compared with the positive control indomethacin at 1 μM.

#### 2.2.6. Effects of Compounds **A2**, **A6**, **B7** on iNOS and COX-2 Expression Levels

**Figure 6:**
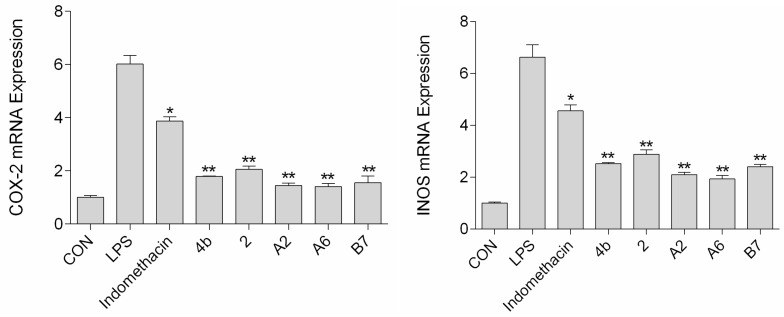
The expression of COX-2 and iNOS mRNA after treatment with 10 μM compound **4b**, **2**, **A2**, **A6**, **B7**, or indomethacin (*: *p* < 0.05; **: *p* < 0.01).

**Figure 7:**
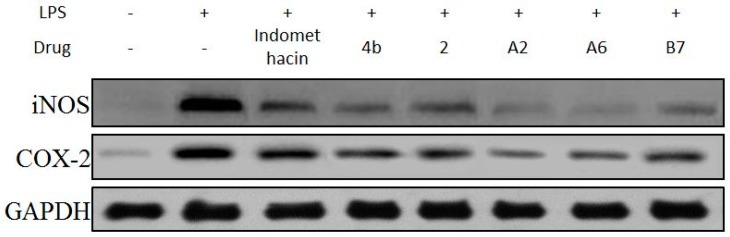
Immunoblotting of cellular proteins COX-2 and iNOS expression in RAW264.7 cells treated with 10 μM compounds **4b**, **2**, **A2**, **A6**, **B7**, or indomethacin. GAPDH was used as a loading control.

The effects of compound **A2**, **A6**, and **B7** treatment on mRNA and protein expression of iNOS and COX-2 in RAW264.7 cells were investigated by RT-PCR analysis and Western blotting. As shown in Figure 6, treatment with **A2**, **A6**, and **B7** markedly reduced iNOS and COX-2 mRNA levels, with higher inhibitory effects compared to indomethacin. Moreover, as shown in Figure 7, compounds **A2**, **A6**, and **B7** significantly decreased the protein levels of iNOS and COX-2.

#### 2.2.7. Effects of Compounds **A2**, **A6**, **B7** on Cellular NF-κB p65 Translocation

**Figure 8:**
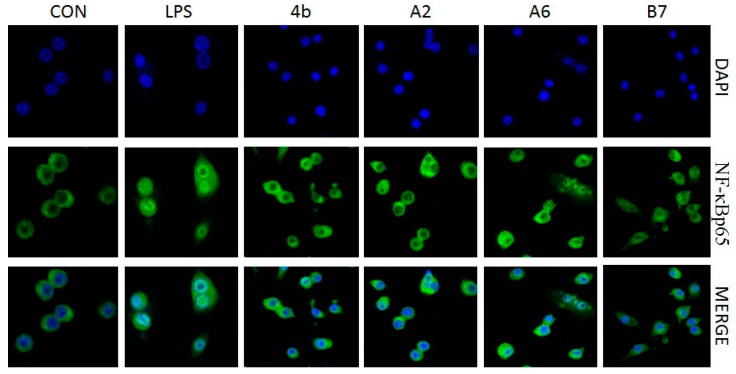
Immunofluorescence of NF-κB p65 in RAW264.7 cells exposed to LPS (100 ng/mL) with or without compound **4b**, **A2**, **A6**, and **B7** (10 μM).

Transcription factor NF-κB signaling is pivotal in the induction of inflammatory responses. A key event involves IκB (inhibitors of NF-kB) phosphorylation and degradation that leads to the release of NF-*κ*B p65 subunit from the cytoplasm, followed by translocation to the nucleus, where it binds to target promoters, and activates transcription of inflammatory genes including TNF-α, IL-6, IL-1β, IL-12, and COX-2. The effects of compound **A2**, **A6**, **B7** on NF-κB p65 subunit nuclear translocation was determined in an immunofluorescence assay. As shown in Figure 8, LPS induction increased NF-κB p65 nuclear translocation (green dots in blue nucleus), while, in **A2**, **A6**, and **B7** pretreated macrophages, LPS-induced nuclear p65 decreased, suggesting that **A2**, **A6**, and **B7** inhibited p65 translocation from cytoplasm to nuclei. The results suggest that the anti-inflammatory activity of compounds **A2**, **A6**, and **B7** may be associated with inhibitory effects on NF-κB activation.

#### 2.2.8. Effects of Compounds **A2**, **A6**, **B7** on MAPKs Phosphorylation in LPS-Stimulated RAW264.7 Cells

**Figure 9:**
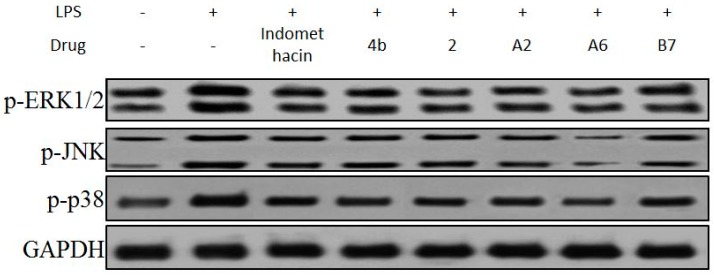
Immunoblotting of p-ERK1/2, p-p38, and p-JNK of MAPK signaling pathway in RAW264.7 cells treated with 10 μM compound **4b**, **2**, **A2**, **A6**, and **B7**, or indomethacin. GAPDH was used as a loading control.

In mitogen-activated protein kinase (MAPK) pathways, ERK1/2, p38, and JNK protein kinases are essential regulators of the inflammatory response [46]. The effects of compound **A2**, **A6**, **B7** on the activation of MAPKs were determined by Western blot analysis using specific antibodies for the corresponding phosphorylated forms. As displayed in Figure 9, compound **A2**, **A6**, and **B7** treatment markedly inhibited the phosphorylation of ERK1/2, p38, and JNK compared to indomethacin treatment, with compound **A6** exhibiting the most significant inhibitory effect.

#### 2.2.9. Effects of Compounds **A2**, **A6**, **B7** Treatment on NF-κB Activation in LPS-Stimulated RAW264.7 Cells

**Figure 10:**
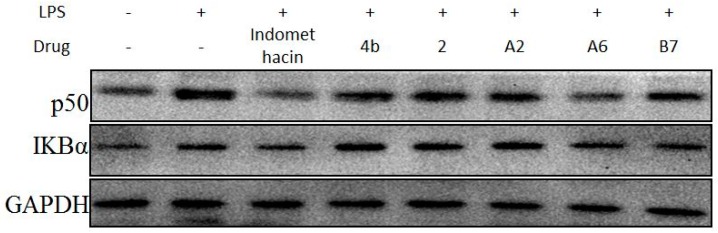
Immunoblotting of p50 and IKB*α* of NF-*κ*B signaling pathway in RAW264.7 cells treated with 10 μM compound **4b**, **2**, **A2**, **A6**, **B7**, or indomethacin. GAPDH was used as a loading control.

Due to the importance of NF-*κ*B activation in the regulation of the inflammatory response, the effects of compound **A2**, **A6**, and **B7** treatment on LPS-induced changes in the levels of p50 and I*κ*B*α* were determined (Figure 10). The results showed that, similar to indomethacin, treatment with **A2**, **A6**, and **B7** effectively blocked LPS-induced activation of NF-*κ*B in macrophages, with **A6** exhibiting the most significant inhibitory effect.

#### 2.2.10. Anti-Inflammatory Activity of **A2**, **A6**, and **B7** in Carrageenan-Induced Rat Paw Edema

**Figure 11:**
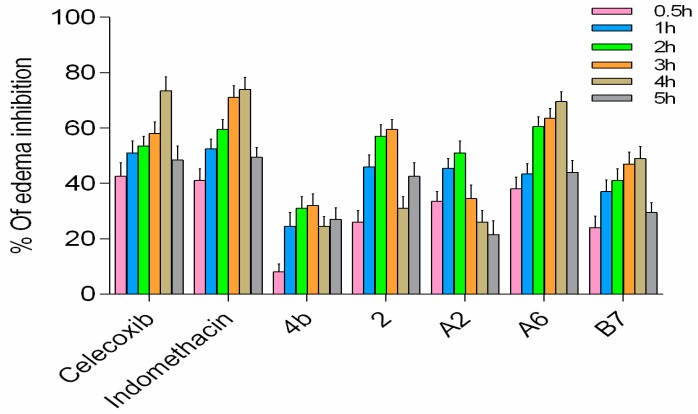
In vivo anti-inflammatory activity of **A2**, **A6**, and **B7** against carrageenan-induced rat paw edema.

In the rat paw edema model, both the compound group and positive control group showed reduction of carrageenan-induced rat paw edema at varying levels (Figure 11). Compounds **A2**, **A6**, and **B7** displayed improved activity compared to **4b**, with **A6** exhibiting the highest in vivo activity among the three new analogs, comparable to celecoxib and indomethacin. Based on all results, **A6** can serve as a lead compound for further studies.

## 3. Materials and Methods

### 3.1. General

Melting points were determined in SGWX-4 microscopic melting point meter and are uncorrected. ^1^H- and ^13^C-NMR spectra were recorded on Bruker 600 MHz NMR spectrometer, using CDCl_3_ or DMSO-d_6_ as solvents. Chemical shifts are expressed in ppm with TMS as internal reference. *J* values are provided in hertz. Mass spectra were recorded on Bruker micrOTOF QII mass spectrometer. Reactions were monitored by thin layer chromatography (TLC) on glass plates coated with silica gel GF-254. Column chromatography was performed with 200–300 mesh silica gel.

#### 3.1.1. General Procedure for the Synthesis of **1**

To a round bottom flask, 3.2 g sulfur was added, along with 11 mL cyanoacetic acid ethyl ester, 10 mL cyclohexanone, and 20 mL ethanol at room temperature. The mixture was stirred for 10 min, then 8 mL diethylamine was added and the reaction was stirred at room temperature for 12 h. The reaction mixture was suction-filtered and the cake was rinsed with water:ethanol = 1:1 three times, and the filtrate was dried to afford a light yellow solid.

#### 3.1.2. General Procedure for the Synthesis of **A1**–**6**

One gram of intermediate **1** and **2** molar equivalents of an appropriately substituted nitrile were dissolved in 15 mL dioxane, and the mixture was stirred at 80 °C for 10 min, then 20 mL hydrochloric acid was added. The reaction continued for 6 h, and was cooled to ambient temperature, poured into ice water, and adjusted to slightly basic using ammonia. The precipitate was suction-filtered to afford the crude product. Silica gel column chromatography using 3:1 petroleum ether:ethyl acetate as eluent yielded compounds **A1**–**6**.

#### 3.1.3. General Procedure for the Synthesis of **2** and **3**

One gram of intermediate **1** and 0.1 g of acetic anhydride were dissolved in triethoxymethane, and the reaction was refluxed for 6 h. The reaction was cooled to ambient temperature and concentrated under vacuum to give a red semi-solid. Recrystallization of the crude product using ethanol/*n*-hexane afforded intermediate **2**. Intermediate **2** was added to hydrazine hydrate in a molar ratio of 1:1.5, and the neat reaction was refluxed overnight, and monitored by TLC until completion. TLC analysis was performed using 1:1 petroleum ether:ethyl acetate, and the retention factor of target materials are 0.4–0.6. The reaction mixture was concentrated under vacuum, and the crude product was recrystallized using 1:1 ethanol/*n*-hexane to afford Intermediates **3**.

#### 3.1.4. General Procedure for the Synthesis of **B1**–**20**

To a round bottom flask, 1.12 g of intermediate **3** was added, along with 6 mL anhydrous ethanol, and appropriately substituted aldehyde. The reaction was warmed to 50 °C and 2 mL acetic acid was added. The reaction was refluxed for 6 h, and then the response process was monitored by TLC analysis using 1:2 petroleum ether:ethyl acetate, and the retention factor of target compounds are 0.3–0.5. The reaction mixture was cooled to room temperature and was filtered. The product was purified by crystallization with ethanol to afford compounds **B1**–**20**.

*2-Phenyl-5,6,7,8-tetrahydrobenzo[4,5]thieno[2,3-d]pyrimidin-4(3H)-one* (**A1**). Yield 79%; m.p. 210–212 °C; ^1^H-NMR (600 MHz, CDCl_3_): δ 8.40 (d, *J* = C-NMR 7.8 Hz, 2H, 2’,6’-Ph-H), 7.44 (dt, *J*_1_ = 7.8 Hz, *J*_2_ = 6.0 Hz, 3H, 3’,4’,5’-Ph-H), 5.34 (s, 1H, -NH), 2.95 (t, *J* = 5.4 Hz, 2H, 8-Tetrahydrophenyl-H), 2.83 (t, *J* = 4.8 Hz, 2H, 5-Tetrahydrophenyl-H), 1.96 (m, 4H, 6,7-Tetrahydrophenyl-H); ^13^C-NMR (151 MHz, CDCl_3_) δ 163.37, 158.69, 145.76, 137.40, 134.71, 131.18, 129.18, 127.05, 113.52, 25.73, 25.28, 22.57, 22.51; ESI-MS *m*/*z*: 283.2 [M + 1]^+^, calculated for C_16_H_14_N_2_OS: 282.4.

*2-(Pyridin-2-yl)-5,6,7,8-tetrahydrobenzo[4,5]thieno[2,3-d]pyrimidin-4(3H)-one* (**A2**). Yield 80%; m.p. 201–203 °C; ^1^H-NMR (600 MHz, CDCl_3_) δ 8.82 (d, *J* = 3.6 Hz, 1H, Pyridin-H), 8.50 (d, *J* = 7.8 Hz, 1H, Pyridin-H), 7.84 (t, *J* = 7.8 Hz, 1H, Pyridin-H), 7.43 (m, 1H, Pyridin-H), 5.85 (s, 1H, -NH), 2.98 (t, *J* = 12.6 Hz, 2H, 8-Tetrahydrophenyl-H), 2.85 (t, *J* = 5.4 Hz, 2H, 5-Tetrahydrophenyl-H), 1.95 (m, 4H, 6,7-Tetrahydrophenyl-H); ^13^C-NMR (151 MHz, CDCl_3_) δ 163.58, 158.08, 149.00, 148.72, 148.21, 137.61, 134.92, 132.26, 126.06, 123.40, 121.90, 25.77, 25.51, 23.11, 22.38. ESI-MS *m*/*z*: 284.2 [M + 1]^+^, calculated for C_15_H_13_N_3_OS: 283.4.

*2-(Pyrimidin-2-yl)-5,6,7,8-tetrahydrobenzo[4,5]thieno[2,3-d]pyrimidin-4(3H)-one* (**A3**). Yield 82%; m.p. 220–221°C. ^1^H-NMR (600 MHz, CDCl_3_) δ 10.78 (s, 1H, -NH), 8.96 (d, *J* = 4.8 Hz, 2H, 4’,6’-Pyrimidin-H), 7.47 (t, *J* = 4.8 Hz, 1H, 5-Pyrimidin-H), 3.09 (t, *J* = 5.4 Hz, 2H, 8-Tetrahydrophenyl-H), 2.84 (t, *J* = 5.4 Hz, 2H, 5-Tetrahydrophenyl-H), 1.95 (m, 4H, 6,7-Tetrahydrophenyl-H); ^13^C-NMR (151 MHz, CDCl_3_) δ 167.76, 162.90, 158.24, 158.08, 156.48, 136.88, 125.73, 121.00, 116.32, 26.22, 25.78, 22.65, 22.61; ESI-MS *m*/*z*: 285.2 [M + 1]^+^, calculated for C_14_H_12_N_4_OS: 284.3.

*2-(4-Hydroxyphenyl)-5,6,7,8-tetrahydrobenzo[4,5]thieno[2,3-d]pyrimidin-4(3H)-one* (**A4**). Yield 64%; m.p. > 250 °C. ^1^H-NMR (600 MHz, DMSO-*d*_6_) δ 12.21 (s, 1H, -OH), 10.28 (s, 1H, -NH), 7.98 (d, *J* = 9.0 Hz, 2H, 2’,6’-Ph-H), 6.86 (d, *J* = 9.0 Hz, 2H, 3’,5’-Ph-H), 2.88 (t, *J* = 6.0 Hz, 2H, 8-Tetrahydrophenyl-H), 2.71 (t, *J* = 6.0 Hz, 2H, 5-Tetrahydrophenyl-H), 1.77 (m, 4H, 6,7-Tetrahydrophenyl-H); ^13^C-NMR (151 MHz, DMSO-*d*_6_) δ163.34, 158.67, 157.96, 145.73, 137.40, 130.72, 127.54, 127.33, 116.47, 114.64, 26.07, 25.69, 22.71, 22.68; ESI-MS *m*/*z*: 298.9 [M + 1]^+^, calculated for C_16_H_14_N_2_O_2_S: 298.3.

*2-(4-Aminophenyl)-5,6,7,8-tetrahydrobenzo[4,5]thieno[2,3-d]pyrimidin-4(3H)-one* (**A5**). Yield 66%; m.p. > 250 °C. ^1^H-NMR (600 MHz, DMSO-*d*_6_) δ 8.03 (d, *J* = 9.0 Hz, 2H, 2’,6’-Ph-H), 6.82(s, 1H, -NH), 6.59 (m, 4H, 3’,5’-Ph-H + NH_2_), 2.88 (t, 2H, *J* = 6.0 Hz, 8-Tetrahydrophenyl-H), 2.71 (t, 2H, *J* = 6.0 Hz, 5-Tetrahydrophenyl-H), 1.80 (m, 4H, 6,7-Tetrahydrophenyl-H); ^13^C-NMR (151 MHz, DMSO-*d*_6_) δ166.64, 158.77, 157.96, 150.71, 131.40, 130.02, 129.17, 127.04, 125.03, 113.42, 112.84, 26.23, 25.78, 22.65, 22.61; ESI-MS *m*/*z*: 298.2 [M + 1]^+^, calculated for C_16_H_15_N_3_OS: 297.4.

*2-(Chloromethyl)-5,6,7,8-tetrahydrobenzo[4,5]thieno[2,3-d]pyrimidin-4(3H)-one* (**A6**). Yield 79%; m.p. 234–236 °C. ^1^H-NMR (600 MHz, DMSO-*d*_6_) δ 4.51 (s, 2H, 2-CH_2_Cl), 2.84 (t, *J* = 5.4 Hz, 2H, 8-Tetrahydrophenyl-H), 2.72 (t, *J* = 4.8 Hz, 2H, 5-Tetrahydrophenyl-H), 1.75 (m, 4H, 6,7-Tetrahydrophenyl-H); ^13^C-NMR (151 MHz, DMSO-*d*_6_) δ 162.26, 158.53, 152.16, 133.74, 131.14, 121.86, 42.86, 25.44, 24.75, 22.66, 21.94. ESI-MS *m*/*z*: 254.8 [M + 1]^+^, calculated for C_11_H_11_ClN_2_OS: 254.7.

*(E)-3-(Benzylideneamino)-5,6,7,8-tetrahydrobenzo[4,5]thieno[2,3-d]pyrimidin-4(3H)-one* (**B1**). Yield 83%; m.p. 216–218 °C. ^1^H-NMR (600 MHz, CDCl_3_) δ 9.67 (s, 1H, N=CH-Ph), 8.19 (s, 1H, 2-pyrimidin-H), 7.89 (dd, *J*_1_ = 7.8 Hz, *J*_2_ = 1.8 Hz, 1H, Ph-H), 7.36 (m, 1H, Ph-H), 7.09 (t, *J* = 7.2 Hz, 1H, Ph-H), 6.95 (d, *J* = 8.4 Hz, 1H, Ph-H), 3.04 (t, *J* = 6.0 Hz, 2H, 8-Tetrahydrophenyl-H), 2.77 (t, *J* = 6.0 Hz, 2H, 5-Tetrahydrophenyl-H), 1.91 (m, 4H, 6,7-Tetrahydrophenyl-H); ^13^C-NMR (151MHz, CDCl_3_) δ 161.39, 160.58, 159.15, 156.69, 147.36, 134.57, 134.13, 132.30, 127.01, 123.49, 120.07, 119.99, 115.23, 25.87, 25.46, 23.00, 22.40. ESI-MS *m*/*z*: 310.2 [M + 1]^+^, calculated for C_17_H_15_N_3_OS: 309.4.

*(E)-3-((2-Methoxybenzylidene)amino)-5,6,7,8-tetrahydrobenzo[4,5]thieno[2,3-d]pyrimidin-4(3H)-one* (**B2**). Yield 80%; m.p. 169–171 °C. ^1^H-NMR (600 MHz, CDCl_3_) δ 9.69 (s, 1H, N=CH-Ph), 8.19 (s, 1H, 2-pyrimidin-H), 8.09 (d, *J* = 7.8 Hz, 1H, Ph-H), 7.48 (m, 1H, Ph-H), 7.04 (t, *J* = 7.8 Hz, 1H, Ph-H), 6.95 (d, *J* = 8.4 Hz, 1H, Ph-H), 3.88 (s, 3H, -OCH_3_), 3.05 (t, *J* = 6.0 Hz, 2H, 8-Tetrahydrophenyl-H), 2.79 (t, *J* = 6.0 Hz, 2H, 5-Tetrahydrophenyl-H), 1.91 (m, 4H, 6,7-Tetrahydrophenyl-H); ^13^C-NMR (151 MHz, CDCl_3_) δ 161.49, 160.62, 159.65, 156.09, 144.89, 134.65, 134.03, 132.30, 127.19, 123.49, 121.39, 120.99, 111.40, 55.77, 25.87, 25.46, 23.00, 22.40; ESI-MS *m*/*z*: 340.2 [M + 1]^+^, calculated for C_18_H_17_N_3_O_2_S: 339.4.

*(E)-3-((2,3-Dimethoxybenzylidene)amino)-5,6,7,8-tetrahydrobenzo[4,5]thieno[2,3-d]pyrimidin-4(3H)-one* (**B3**). Yield 78%; m.p. 226–228 °C. ^1^H-NMR (600 MHz, CDCl_3_) δ 9.66 (s, 1H, N=CH-Ph), 8.19 (s, 1H, 2-pyrimidin-H), 7.68 (dd, *J*_1_ = 7.8 Hz, *J*_2_ = 1.2 Hz, 1H, Ph-H), 7.14 (t, *J* = 7.8 Hz, 1H, Ph-H), 7.07 (dd, *J*_1_ = 8.4 Hz, *J*_2_ = 1.2 Hz, 1H, Ph-H), 3.94 (s, 3H, -OCH_3_), 3.91 (s, 3H, -OCH_3_), 3.06 (t, *J* = 6.0 Hz, 2H, 8-Tetrahydrophenyl-H), 2.80 (t, *J* = 6.0 Hz, 2H, 5-Tetrahydrophenyl-H), 1.91 (m, 4H, 6,7-Tetrahydrophenyl-H); ^13^C-NMR (151 MHz, CDCl_3_) δ 161.31, 160.60, 156.09, 153.04, 150.47, 144.98, 134.72, 132.36, 126.85, 124.44, 123.50, 118.43, 116.12, 62.34, 56.10, 25.92, 25.48, 23.01, 22.40. ESI-MS *m*/*z*: 370.2 [M + 1]^+^, calculated for C_19_H_19_N_3_O_3_S: 369.4.

*(E)-3-((2-Hydroxy-3-methoxybenzylidene)amino)-5,6,7,8-tetrahydrobenzo[4,5]thieno[2,3-d]pyrimidin-4(3H)-one* (**B4**). Yield 82%; m.p. 197–199 °C. ^1^H-NMR (600 MHz, CDCl_3_) δ 10.43 (s, 1H, -OH), 9.42 (s, 1H, N=CH-Ph), 8.13 (s, 1H, 2-Pyrimidin-H), 7.05 (m, 2H, Ph-H), 6.94 (t, *J* = 7.8 Hz, 1H, Ph-H), 3.94 (s, 3H, -OCH_3_), 3.03 (t, *J* = 6.0 Hz, 2H, 8-Tetrahydrophenyl-H), 2.80 (t, *J* = 6.0 Hz, 2H, 5-Tetrahydrophenyl-H), 1.91 (m, 4H, 6,7-Tetrahydrophenyl-H); ^13^C-NMR (151 MHz, CDCl_3_) δ 161.31, 160.60, 156.09, 153.04, 150.47, 144.98, 134.72, 132.36, 126.85, 124.44, 123.50, 118.43, 116.12, 62.34, 25.92, 25.48, 23.01, 22.40. ESI-MS *m*/*z*: 356.2 [M + 1]^+^, calculated for C_18_H_17_N_3_O_3_S: 355.4.

*(E)-3-((2-Nitrobenzylidene)amino)-5,6,7,8-tetrahydrobenzo[4,5]thieno[2,3-d]pyrimidin-4(3H)-one* (**B5**). Yield 69%; m.p. 225–227 °C. ^1^H-NMR (600 MHz, CDCl_3_) δ 10.17 (s, 1H, N=CH-Ph), 8.24 (s, 1H, 2-Pyrimidin-H), 8.15 (m, 2H, Ph-H), 7.77 (t, *J* = 7.8 Hz, 1H, Ph-H), 7.71 (m, 1H, Ph-H), 3.04 (t, *J* = 6.0 Hz, 2H, 8-Tetrahydrophenyl-H), 2.81 (t, *J* = 6.0 Hz, 2H, 5-Tetrahydrophenyl-H), 1.91 (m, 4H, 6,7-Tetrahydrophenyl-H); ^13^C-NMR (151 MHz, CDCl_3_) δ 160.49, 159.73, 156.43, 148.86, 145.36, 133.95, 132.55, 132.51, 132.12, 129.62, 128.83, 125.05, 123.52, 25.83, 25.48, 22.97, 22.35. ESI-MS *m*/*z*: 355.2 [M + 1]^+^, calculated for C_17_H_14_N_4_O_3_S: 354.4.

*(E)-3-((2-(Trifluoromethyl)benzylidene)amino)-5,6,7,8-tetrahydrobenzo[4,5]thieno[2,3-d]pyrimidin-4(3H)-one* (**B6**). Yield 76%; m.p. 239–241°C. ^1^H-NMR (600 MHz, CDCl_3_) δ 10.17 (s, 1H, N=CH-Ph), 8.24 (s, 1H, 2-Pyrimidin-H), 8.15 (m, 2H, Ph-H), 7.77 (t, *J* = 7.8 Hz, 1H, Ph-H), 7.71 (m, 1H, Ph-H), 3.04 (t, *J* = 6.0 Hz, 2H, 8-Tetrahydrophenyl-H), 2.81 (t, *J* = 6.0 Hz, 2H, 5-Tetrahydrophenyl-H), 1.91 (m, 4H, 6,7-Tetrahydrophenyl-H); ^13^C-NMR (151 MHz, CDCl_3_) δ 160.36, 159.26, 156.51, 145.56, 135.08, 132.56, 132.29, 131.57, 131.48, 127.90, 126.18, 124.86, 123.51, 25.91, 25.47, 22.98, 22.34; ESI-MS *m*/*z*: 378.1 [M + 1]^+^, C_18_H_14_F_3_N_3_OS: 377.4.

*(E)-3-((Naphthalen-2-ylmethylene)amino)-5,6,7,8-tetrahydrobenzo[4,5]thieno[2,3-d]pyrimidin-4(3H)-one* (**B7**). Yield 81%; m.p. > 250 °C. ^1^H-NMR (600 MHz, CDCl_3_) δ 9.71 (s, 1H, N=CH-Ph), 8.28 (s, 1H, 2-Pyrimidin-H), 8.15 (s, 1H, Naphthaline-H), 8.09 (dd, *J*_1_ = 8.4 Hz, *J*_2_ = 1.2 Hz, 1H, Naphthaline-H), 7.91 (d, *J* = 8.4 Hz, 2H, Naphthaline-H), 7.88 (d, *J* = 7.8 Hz, 1H, Naphthaline-H), 7.60–7.53 (m, 2H, Naphthaline-H), 3.06 (t, *J* = 6.0 Hz, 2H, 8-Tetrahydrophenyl-H), 2.80 (t, *J* = 6.0 Hz, 2H, 5-Tetrahydrophenyl-H), 1.93 (m, 4H, 6,7-Tetrahydrophenyl-H); ^13^C-NMR (151 MHz, CDCl_3_) δ 163.85, 160.54, 156.61, 145.43, 135.45, 134.96, 133.10, 132.27, 130.86, 129.02, 128.14, 126.99, 123.48, 122.88, 25.87, 25.47, 22.99, 22.40. ESI-MS *m*/*z*: 360.2 [M + 1]^+^, calculated for C_21_H_17_N_3_OS: 359.4.

*(E)-3-(((1-Methyl-1H-indol-2-yl)methylene)amino)-5,6,7,8-tetrahydrobenzo[4,5]thieno[2,3-d]pyrimidin-4(3H)-one* (**B8**). Yield 78%; m.p. 247–249 °C; ^1^H-NMR (600 MHz, CDCl_3_) δ 9.25 (s, 1H, N=CH-Ph), 8.35 (d, *J* = 7.8 Hz, 1H, Benzazole-H), 8.24 (s, 1H, 2-Pyrimidin-H), 7.48 (s, 1H, Benzazole-H), 7.36 (dd, *J*_1_ = 4.8 Hz, *J*_2_ = 1.2 Hz, 2H, Benzazole-H), 7.31 (m, 1H, Benzazole-H), 3.82 (s, 3H, N-CH_3_), 3.05 (t, *J* = 6.0 Hz, 2H, 8-Tetrahydrophenyl-H), 2.79 (t, *J* = 6.0 Hz, 2H, 5-Tetrahydrophenyl-H), 1.87 (m, 4H, 6,7-Tetrahydrophenyl-H); ^13^C-NMR (151 MHz, CDCl_3_) δ 161.44, 160.72, 156.36, 144.89, 138.23, 136.33, 134.33, 132.12, 125.39, 123.84, 123.50, 123.14, 122.27, 110.58, 109.87, 33.57, 25.87, 25.46, 23.02, 22.43; ESI-MS *m*/*z*: 363.3 [M + 1]^+^, calculated for C_20_H_18_N_4_OS: 362.4.

*(E)-3-((Furan-2-ylmethylene)amino)-5,6,7,8-tetrahydrobenzo[4,5]thieno[2,3-d]pyrimidin-4(3H)-one* (**B9**). Yield 78%; m.p. > 250 ° C. ^1^H-NMR (600 MHz, CDCl_3_) δ 9.52 (s, 1H, N=CH-Ph), 8.24 (s, 1H, 2-Pyrimidin-H), 7.66 (d, *J* = 1.8 Hz, 1H, Furan-H), 7.05 (m, 1H, Furan-H), 6.59 (dd, *J*_1_ = 3.6 Hz, *J*_2_ = 1.8 Hz, 1H, Furan-H), 3.03 (t, *J* = 6.0 Hz, 2H, 8-Tetrahydrophenyl-H), 2.79 (t, *J* = 6.0 Hz, 2H, 5-Tetrahydrophenyl-H), 1.95 (m, 4H, 6,7-Tetrahydrophenyl-H); ^13^C-NMR (151 MHz, CDCl_3_) δ 160.47, 156.81, 151.58, 148.36, 146.74, 145.52, 135.10, 132.27, 123.38, 118.76, 112.64, 25.84, 25.47, 22.97, 22.38. ESI-MS *m*/*z*: 300.1 [M + 1]^+^, calculated for C_15_H_13_N_3_O_2_S:299.4.

*3-((E)-((E)-3-(Furan-2-yl)allylidene)amino)-5,6,7,8-tetrahydrobenzo[4,5]thieno[2,3-d]pyrimidin-4(3H)-one* (**B10**). Yield 81%; m.p. 218–220 °C; ^1^H-NMR (600 MHz, CDCl_3_) δ 9.18 (d, *J* = 9.0 Hz, 1H, N=CH-Ph), 8.13 (s, 1H, 2-Pyrimidin-H), 7.52 (d, *J* = 1.8 Hz, 1H, Furan-H), 6.94 (d, *J* = 15.6 Hz, 1H, CH=CH), 6.88 (dd, *J*_1_ = 15.6 Hz, *J*_2_ = 9.0 Hz, 1H, CH=CH), 6.59 (d, *J* = 3.6 Hz, 1H, Furan-H), 6.49 (dd, *J*_1_ = 3.6 Hz, *J*_2_ = 1.8 Hz, 1H, Furan-H), 3.03 (t, *J* = 6.0 Hz, 2H, 8-Tetrahydrophenyl-H), 2.79 (t, *J* = 6.0 Hz, 2H, 5-Tetrahydrophenyl-H), 1.91 (m, 4H, 6,7-Tetrahydrophenyl-H); ^13^C-NMR (151 MHz, CDCl_3_) δ 165.51, 160.54, 156.45, 151.73, 145.10, 144.88, 134.90, 132.27, 131.82, 123.42, 122.52, 113.95, 112.56, 25.86, 25.47, 22.99, 22.40; ESI-MS *m*/*z*: 326.1 [M + 1]^+^, calculated for C_17_H_15_N_3_O_2_S: 325.4.

*(E)-3-((4-(Benzyloxy)benzylidene)amino)-5,6,7,8-tetrahydrobenzo[4,5]thieno[2,3-d]pyrimidin-4(3H)-one* (**B11**). Yield 71%; m.p. > 250 °C; ^1^H-NMR (600 MHz, CDCl_3_) δ 9.32 (s, 1H, N=CH-Ph), 8.18 (s, 1H, 2-Pyrimidin-H), 7.79 (dd, *J*_1_ = 19.2 Hz, *J*_2_ = 8.4 Hz, 2H, Ph-H), 7.47 (m, 5H, Ph-H), 7.04 (dd, *J*_1_ = 12.6 Hz, *J*_2_ = 9.0 Hz, 2H, Ph-H), 5.13 (d, *J* = 9.6 Hz, 2H, -CH_2_-), 3.04 (t, *J* = 6.0 Hz, 2H, 8-Tetrahydrophenyl-H), 2.79 (t, *J* = 6.0 Hz, 2H, 5-Tetrahydrophenyl-H), 1.95 (m, 4H, 6,7-Tetrahydrophenyl-H); ^13^C-NMR (151 MHz, CDCl_3_) δ 164.26, 162.32, 156.40, 146.16, 145.07, 136.38, 134.80, 130.71, 130.28, 128.84, 128.38, 127.64, 125.87, 123.45, 115.41,115.28, 70.32, 25.86, 25.47, 22.99, 22.40. ESI-MS *m*/*z*: 416.3 [M + 1]^+^, calculated for C_24_H_21_N_3_O_2_S: 415.5.

*(E)-3-((4-(Dimethylamino)benzylidene)amino)-5,6,7,8-tetrahydrobenzo[4,5]thieno[2,3-d]pyrimidin-4(3H)-one* (**B12**). Yield 83%; m.p. 209–211 °C; ^1^H-NMR (600 MHz, CDCl_3_) δ 8.99 (s, 1H, N=CH-Ph), 8.16 (s, 1H, 2-Pyrimidin-H), 7.72 (d, *J* = 9.0 Hz, 2H, Ph-H), 6.71 (d, *J* = 9.0 Hz, 2H, Ph-H), 3.07 (s, 6H, N-CH_3_), 3.05–3.03 (t, *J* = 6.0 Hz, 2H, 8-Tetrahydrophenyl-H), 2.79 (t, *J* = 6.0 Hz, 2H, 5-Tetrahydrophenyl-H), 1.90 (m, 4H, 6,7-Tetrahydrophenyl-H); ^13^C-NMR (151 MHz, CDCl_3_) δ 166.14, 160.70, 156.27, 153.33, 146.16, 144.70, 134.45, 132.18, 130.78, 123.46, 119.98, 111.64, 111.46, 40.23, 25.87, 25.48, 23.03, 22.43. ESI-MS *m*/*z*: 353.2 [M + 1]^+^, calculated for C_19_H_20_N_4_OS: 352.4.

*(E)-3-((4-Isopropylbenzylidene)amino)-5,6,7,8-tetrahydrobenzo[4,5]thieno[2,3-d]pyrimidin-4(3H)-one* (**B13**). Yield 79%; m.p. 214–216 °C; ^1^H-NMR (600 MHz, CDCl_3_) δ 9.46 (s, 1H, N=CH-Ph), 8.21 (s, 1H, 2-Pyrimidin-H), 7.78 (d, *J* = 8.4 Hz, 2H, Ph-H), 7.33 (d, *J* = 8.4 Hz, 2H, Ph-H), 3.04 (t, *J* = 6.0 Hz, 2H, 8-Tetrahydrophenyl-H), 2.98 (td, *J*_1_ = 13.8 Hz, *J*_2_ = 7.2 Hz, 1H, CH), 2.79 (t, *J* = 6.0 Hz, 2H, 5-Tetrahydrophenyl-H), 1.93 (m, 4H, 6,7-Tetrahydrophenyl-H), 1.28 (d, *J* = 7.2 Hz, 6H, CH_3_); ^13^C-NMR (151 MHz, CDCl_3_) δ 164.29, 160.56, 156.49, 153.94, 145.28, 134.82, 132.25, 130.77, 128.94, 127.20, 123.46, 34.44, 25.86, 25.46, 23.86, 22.98, 22.39; ESI-MS *m*/*z*: 352.3 [M + 1]^+^, calculated for C_20_H_21_N_3_OS:351.4.

*(E)-3-((4-(Tert-butyl)benzylidene)amino)-5,6,7,8-tetrahydrobenzo[4,5]thieno[2,3-d]pyrimidin-4(3H)-one* (**B14**). Yield 85%; m.p. 230–232 °C; ^1^H-NMR (600 MHz, CDCl_3_) δ 9.47 (s, 1H, N=CH-Ph), 8.21 (s, 1H, 2-Pyrimidin-H), 7.79 (d, *J* = 8.4 Hz, 2H, Ph-H), 7.50 (d, *J* = 8.4 Hz, 2H, Ph-H), 3.05 (m, 2H, 8-Tetrahydrophenyl-H), 2.80 (m, 2H, 5-Tetrahydrophenyl-H), 1.94 (m, 4H, 6,7-Tetrahydrophenyl-H), 1.36 (s, 9H, CH_3_); ^13^C-NMR (151 MHz, CDCl_3_) δ 164.18, 160.02, 156.53, 156.19, 145.33, 134.85, 132.27, 130.41, 128.67, 126.07, 123.49, 112.29, 35.28, 31.28, 25.88, 25.48, 23.00, 22.40; ESI-MS *m*/*z*: 366.3 [M + 1]^+^, calculated for C_21_H_23_N_3_OS: 365.4.

*(E)-3-((2,6-Difluorobenzylidene)amino)-5,6,7,8-tetrahydrobenzo[4,5]thieno[2,3-d]pyrimidin-4(3H)-one* (**B15**). Yield 78%; m.p. 218–220 °C; ^1^H-NMR (600 MHz, CDCl_3_) δ 9.96 (d, *J* = 1.8 Hz, 1H, N=CH-Ph), 8.23 (s, 1H, 2-Pyrimidin-H), 7.78 (m, 1H, Ph-H), 7.22 (m, 1H, Ph-H), 7.13 (m, 1H, Ph-H), 3.05 (t, *J* = 6.0 Hz, 2H, 8-Tetrahydrophenyl-H), 2.80 (t, *J* = 6.0 Hz, 2H, 5-Tetrahydrophenyl-H), 1.94 (m, 4H, 6,7-Tetrahydrophenyl-H); ^13^C-NMR (151 MHz, CDCl_3_) δ 160.39, 156.71, 155.13, 145.68, 135.28, 132.44, 123.45, 113.01, 112.85, 111.48, 25.87, 25.48, 22.97, 22.36; ESI-MS *m*/*z*: 346.2 [M + 1]^+^, calculated for C_17_H_13_F_2_N_3_OS: 345.4.

*(E)-3-((2-Chloro-6-fluorobenzylidene)amino)-5,6,7,8-tetrahydrobenzo[4,5]thieno[2,3-d]pyrimidin-4(3H)-one* (**B16**). Yield 76%; m.p. 226–228 °C; ^1^H-NMR (600 MHz, CDCl_3_) δ 9.99 (s, 1H, N=CH-Ph), 8.22 (s, 1H, 2-Pyrimidin-H), 7.39 (m, 1H, Ph-H), 7.30 (d, *J* = 8.4 Hz, 1H, Ph-H), 7.13 (t, *J* = 9.0 Hz, 1H, Ph-H), 3.06 (t, *J* = 6.0 Hz, 2H, 8-Tetrahydrophenyl-H), 2.80 (t, *J* = 6.0 Hz, 2H, 5-Tetrahydrophenyl-H), 1.92 (m, 4H, 6,7-Tetrahydrophenyl-H); ^13^C-NMR (151 MHz, CDCl_3_) δ 162.25, 160.51, 159.29, 156.66, 145.81, 135.05, 32.88, 132.46, 129.48, 125.43, 123.53, 116.26, 116.11, 25.91, 25.46, 22.97, 22.34; ESI-MS *m*/*z*: 362.2 [M + 1]^+^, calculated for C_17_H_13_ClFN_3_OS: 361.8.

*(E)-3-((2-Bromo-6-fluorobenzylidene)amino)-5,6,7,8-tetrahydrobenzo[4,5]thieno[2,3-d]pyrimidin-4(3H)-one* (**B17**). Yield 71%; m.p. 207–209 °C; ^1^H-NMR (600 MHz, CDCl_3_) δ 9.96 (s, 1H, N=CH-Ph), 8.22 (s, 1H, 2-Pyrimidin-H), 7.49 (d, *J* = 8.4 Hz, 1H, Ph-H), 7.31 (m, 1H, Ph-H), 7.17 (t, *J* = 9.0 Hz, 1H, Ph-H), 3.05 (t, *J* = 6.0 Hz, 2H, 8-Tetrahydrophenyl-H), 2.79 (t, *J* = 6.0 Hz, 2H, 5-Tetrahydrophenyl-H), 1.90 (m, 4H, 6,7-Tetrahydrophenyl-H); ^13^C-NMR (151 MHz, CDCl_3_) δ 162.25, 160.51, 159.29, 156.66, 145.81, 135.05, 32.88, 132.46, 129.48, 125.43, 123.53, 116.26, 116.11, 25.91, 25.46, 22.97, 22.34; ESI-MS *m*/*z*: 406.1, 408.1 [M + 1]^+^, calculated for C_17_H_13_BrFN_3_OS: 406.2.

*(E)-3-((2,5-Dibromobenzylidene)amino)-5,6,7,8-tetrahydrobenzo[4,5]thieno[2,3-d]pyrimidin-4(3H)-one* (**B18**). Yield 75%; m.p. 199–201 °C; ^1^H-NMR (600 MHz, CDCl_3_) δ 10.13 (s, 1H, N=CH-Ph), 8.26 (d, *J* = 2.4 Hz, 1H, Ph-H), 8.25 (s, 1H, 2-Pyrimidin-H), 7.51 (d, *J* = 8.4 Hz, 1H, Ph-H), 7.46 (dd, *J*_1_ = 8.4 Hz, *J*_2_ = 2.4 Hz, 1H, Ph-H), 3.06 (t, *J* = 6.0 Hz, 2H, 8-Tetrahydrophenyl-H), 2.80 (t, *J* = 6.0 Hz, 2H, 5-Tetrahydrophenyl-H), 1.92 (m, 4H, 6,7-Tetrahydrophenyl-H); ^13^C-NMR (151 MHz, CDCl_3_) δ 160.34, 159.81, 156.80, 145.83, 135.84, 135.25, 134.84, 134.52, 132.51, 130.88, 124.68, 123.47, 122.02, 25.94, 25.48, 22.97, 22.34; ESI-MS *m*/*z*: 468.0 [M + 1]^+^, calculated for C_17_H_13_Br_2_N_3_OS: 467.2.

*(E)-4-(((4-Oxo-5,6,7,8-tetrahydrobenzo[4,5]thieno[2,3-d]pyrimidin-3(4H)-yl)imino)methyl)phenyl acetate* (**B19**). Yield 76%; m.p. 215–217 °C; ^1^H-NMR (600 MHz, CDCl_3_) δ 9.60 (s, 1H, N=CH-Ph), 8.22 (s, 1H, 2-Pyrimidin-H), 7.88 (d, *J* = 8.4 Hz, 2H, Ph-H), 7.22 (d, *J* = 8.4 Hz, 2H, Ph-H), 3.05 (t, *J* = 6.0 Hz, 2H, 8-Tetrahydrophenyl-H), 2.80 (t, *J* = 6.0 Hz, 2H, 5-Tetrahydrophenyl-H), 2.33 (s, 3H, COCH_3_), 1.91 (m, 4H, 6,7-Tetrahydrophenyl-H); ^13^C-NMR (151 MHz, CDCl_3_) δ 169.07, 162.43, 156.67, 153.81, 145.49, 135.04, 132.30, 130.90, 130.00, 125.57,122.38, 25.88, 25.48, 22.99, 22.39, 21.32. ESI-MS *m*/*z*: 368.2 [M + 1]^+^, calculated for C_19_H_17_N_3_O_3_S: 367.4.

*(E)-3-((4-(Allyloxy)benzylidene)amino)-5,6,7,8-tetrahydrobenzo[4,5]thieno[2,3-d]pyrimidin-4(3H)-one* (**B20**). Yield 74%; m.p. 178–180 °C; ^1^H-NMR (600 MHz, CDCl_3_) δ 9.32 (s, 1H, N=CH-Ph), 8.19 (s, 1H, 2-Pyrimidin-H), 7.80 (d, *J* = 9.0 Hz, 2H, Ph-H), 6.99 (d, *J* = 9.0 Hz, 2H, Ph-H), 6.07 (m, 1H, CH_2_CH=CH_2_), 5.44 (dd, *J*_1_ = 17.4 Hz, *J*_2_ *=* 1.2 Hz, 1H, CH_2_CH=CH_2_), 5.33 (dd, *J*_1_ = 10.2 Hz, *J*_2_ = 1.2 Hz, 1H, CH_2_CH=CH_2_), 4.61 (m, 2H, CH_2_CH=CH_2_), 3.05 (t, *J* = 6.0 Hz, 2H, 8-Tetrahydrophenyl-H), 2.80 (t, *J* = 6.0 Hz, 2H, 5-Tetrahydrophenyl-H), 1.95 (m, 4H, 6,7-Tetrahydrophenyl-H); ^13^C-NMR (600 MHz, CDCl_3_) δ 164.33, 162.18 160.63, 156.41, 145.08, 134.80, 132.71, 132.24, 130.69, 125.77, 123.47, 118.34, 115.27, 69.07, 25.87, 25.48, 23.01, 22.41. ESI-MS *m*/*z*: 365.1 [M + 1]^+^, calculated for C_20_H_19_N_3_O_2_S: 365.4.

### 3.2. Cell Viability Assay

RAW264.7 cells were plated at 5 × 10^3^/well and cultured in complete RPMI-1640 medium containing 5% heat inactivated serum, 100 U/mL penicillin and 100 mg/mL streptomycin, in incubator under 5% CO_2_ at 37 °C for 24 h. Compounds were dissolved in DMSO and diluted with 1640 medium to the desired concentrations. Cells and compound solutions were incubated for 72 h together, then 5 mg/mL fresh MTT solution was added to every hole and the cells were cultured for another 3 h in the CO_2_ incubator. One hundred milliliters of DMSO was used as blank control, and the optical density was recorded at 590 nm. Cell viability is usually expressed as the ratio of absorbance.

### 3.3. Determination of Cytokines

ELISA was prepared by adding 100 μL/well mixture, which contains 40 μL capture antibodies, 9 mL sterile water, and 1 mL coating buffer, and the plate was kept at 4 °C overnight. On the next day, the plate was washed with PBST three times, sealed for 1 h on table concentrator, and again washed with PBST three times. A mixture of 5% sample and 95% AD was added and the plate was incubated on table concentrator for 2 h. Then 40 μL detection antibody was mixed with 100 μL AD and was added to ELISA plate 100 μL/well and the ELISA plate was incubated on table concentrator for 1 h. The wells were washed with PBST three times and HRP was added at 100 μL/well, incubated on table concentrator for another 30 min. Finally, the wells were washed with PBST five times and TMP was added at 100 μL/well, kept in dark place until color changed to blue. Then, 2 M H_2_SO_4_ was added at 50 μL/well to stop the reaction and the OD value was measured at 450 nm.

### 3.4. Measurement of NO Levels

Ten microliters of cell supernatant was added to 96-well ELISA plate; 100 μL Griess reagent was added and mixed well; and the plate was stewed at room temperature and kept in dark place for 10 min. Absorbance at 540 nm was measured and the content of NO was calculated using the following formula:

Content of NO = $\frac{\mathrm{measured}\;\mathrm{OD}\;\mathrm{value}-\mathrm{blank}\;\mathrm{OD}\;\mathrm{value}}{\mathrm{standard}\;\mathrm{OD}\;\mathrm{value}-\mathrm{blank}\;\mathrm{OD}\;\mathrm{balue}}*\mathrm{standard}\;\mathrm{substance}\;\mathrm{concentration}\left(100\;\mathrm{umol}/L\right)\times \mathrm{diluted}\;\mathrm{multiple}\;\mathrm{of}\;\mathrm{sample}\;\mathrm{before}\;\mathrm{manage}\left(\mathrm{umol}/L\right)$

### 3.5. RNA Isolation and Real-Time Reverse Transcription-Polymerase Chain Reaction (Real-Time RT-PCR)

Ten microliters of 2× Real time PCT Master Mix, 1 μL template, 2 μL primer mixer, and 7 μL 0.1% DEPC water were added in 0.2 mL PCR tube. The conditions of the RT-PCR were as follows: pre-denaturation at 95 °C for 5 min, degeneration at 95 °C for 30 s, annealing 40 cycles at 60 °C for 20 s or 72 °C for 40 s. The primer sequences were as follows:

GAPDH (149 bp)
(Sense primer) 5′ TATGTCGTGGAGTCTACTGGT 3′
(Anti-sense primer) 5′ GAGTTGTCATATTTCTCGTGG 3′
COX2 (74 bp)
(Sense primer) 5′ TGAGCAACTATTCCAAACCAGC 3′
(Anti-sense primer) 5′ GCACGTAGTCTTCGATCACTATC 3′
INOS (127 bp)
(Sense primer) 5′ GTTCTCAGCCCAACAATACAAGA 3′
(Anti-sense primer) 5′ GTGGACGGGTCGATGTCAC 3′

### 3.6. Cellular NF-κB p65 Translocation Assay

The assay was performed using a cellular NF-κB p-65 translocation kit (Beyotime Biotech, Nantong, China) following the manufacturer's instructions. The red and blue fluorescence of the P65 protein and nuclei were visualized simultaneously by fluorescence microscope (Nikon, Tokyo, Japan) at an excitation wavelength of 350 nm for DAPI and 540 nm for Cy3.

### 3.7. Western Blot Analysis

The treated cells were collected and lysed. An amount of 40 μg of the whole cell lysate was separated by 10% sodium dodecyl sulfate−polyacrylamide gel electrophoresis and electro-transferred to a nitrocellulose membrane. Each membrane was pre-incubated for 1 h at room temperature in Tris-buffered saline, pH 7.6, containing 0.05% Tween 20 and 5% non-fat milk. The nitrocellulose membrane was incubated with specific antibodies against p-JNK, IKBα p-p38, p50, p-ERK1/2, COX-2, iNOS, or GAPDH (Santa Cruz Biotech Co. Ltd. Santa Cruz, CA, USA). Immunoreactive bands were detected by incubating with secondary antibody conjugated with horseradish peroxidase and visualized using enhanced chemiluminescence reagents (Bio-Rad, Hercules, CA, USA).

### 3.8. Animal Study

Rats, obtained from Laboratory Animal Center of Wenzhou Medical University (Wenzhou, China), were used to study the anti-inflammatory activity of compounds. All experimental procedures and protocols were reviewed and approved by the Animal Care and Use Committee of Wenzhou Medical University and were in accordance with the Guide for the Care and Use of Laboratory Animals. Forty-eight SD male rats (weighting 150 to 190 g) were divided into 6 groups, with 8 male rats (weighting 150 to 190 g) in each group. Compounds were dissolved in 0.5% aqueous CMC solution. Groups of rats were fed for 3 days in room temperature adaptability conditions. Intragastric administration of 1 mL dose of the compounds was applied at 10 mg/kg per day for three consecutive days. Edema was induced by subcutaneous injection of 1% sterilized carrageenan saline solution in a volume of 0.1 mL/rat at the right hind paws 1 h after the last dose. Rat paw volume was measured after carrageenan injection at 0, 0.5, 1, 2, 3, 4, and 5 h. Measurements were repeated three times and the average was used to assess the extent of paw swelling. The inhibition rate for rat paw swelling was calculated by the following formula:

Inhibition rate (%) = [(V_R_ − V_0_)_control_ − (V_R_ − V_0_)_treat_]/(V_R_ − V_0_)_control_ × 100%

where V_R_ represents the volume of right foot for each group at each time point, and V_0_ represents the volume of the right foot at 0 h.

### 3.9. Statistical Analysis

Data are presented as the mean ± standard error of the mean (SEM). *T*-test was used to analyze the differences between groups of data. Statistical analysis was performed using GraphPad Pro (GraphPad, San Diego, CA, USA). *p* Values less than 0.05 (*p* < 0.05) were considered statistically significant. *p* values less than 0.01 (*p* < 0.01) were considered notable statistically significant. All experiments were repeated at least three times.

## 4. Conclusions

Twenty-six tetrahydrobenzo[4,5]thieno[2,3-*d*]pyrimidine derivatives were synthesized. The anti-inflammatory effects of these compounds were evaluated in a variety of in vitro and in vivo assays, including the inhibition of NO, iNOS, and COX-2 production; pro-inflammatory cytokine secretion; and mRNA expression. In an effort to elucidate the potential mechanisms of anti-inflammatory activity, we also evaluated the phosphorylation of MAPK and the activation of the NF-κB signaling pathway in LPS-stimulated macrophages.

The results of the present study provided evidence that treatment with **A2**, **A6**, and **B7** can inhibit the production of inflammatory mediators, including NO, inflammatory cytokines, iNOS, and COX-2, in LPS-stimulated RAW264.7 cells without cytotoxicity. These effects can be attributed to the inhibition of NF-κB activation by the degradation of IκB*α* and p50 and the blockade of MAPK phosphorylation. Animal experimental findings suggest that the inhibition of carrageenan-induced rat paw edema by **A6** is similar to indomethacin and celecoxib. Taken together, **A6** is selected as a lead compound for further optimization and mechanistic studies.

## Figures and Tables

**Table 1:**
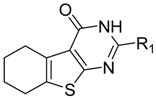
Chemical Structures of Target Compounds **A1**–**6**.

Compound	R_1_	Compound	R_1_
**A1**	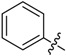	**A4**	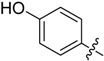
**A2**	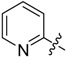	**A5**	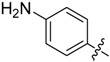
**A3**	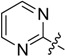	**A6**	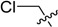

**Table 2:**
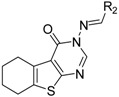
Chemical Structures of Target Compounds **B1**–**20**.

Compound	R_2_	Compound	R_2_
**B1**	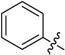	**B11**	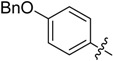
**B2**	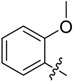	**B12**	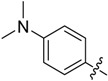
**B3**	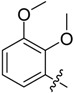	**B13**	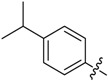
**B4**	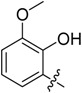	**B14**	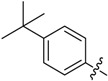
**B5**	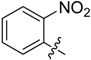	**B15**	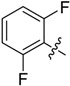
**B6**	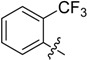	**B16**	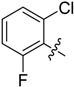
**B7**	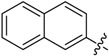	**B17**	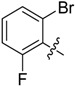
**B8**	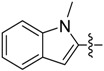	**B18**	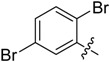
**B9**	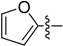	**B19**	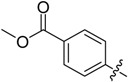
**B10**	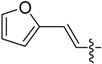	**B20**